# STING: a master regulator in the cancer-immunity cycle

**DOI:** 10.1186/s12943-019-1087-y

**Published:** 2019-11-04

**Authors:** Yuanyuan Zhu, Xiang An, Xiao Zhang, Yu Qiao, Tongsen Zheng, Xiaobo Li

**Affiliations:** 1 Department of Pathology, Harbin Medical University, No. 157 Baojian Road, Nangang District, Harbin, 150081 China; 2 Department of Histology and Embryology, Harbin Medical University, No. 157 Baojian Road, Nangang District, Harbin, 150081 China; 3 Department of Gastrointestinal Medical Oncology, Harbin Medical University Cancer Hospital, No.150 Haping Road, Nangang District, Harbin, 150081 China

## Abstract

The aberrant appearance of DNA in the cytoplasm triggers the activation of cGAS-cGAMP-STING signaling and induces the production of type I interferons, which play critical roles in activating both innate and adaptive immune responses. Recently, numerous studies have shown that the activation of STING and the stimulation of type I IFN production are critical for the anticancer immune response. However, emerging evidence suggests that STING also regulates anticancer immunity in a type I IFN-independent manner. For instance, STING has been shown to induce cell death and facilitate the release of cancer cell antigens. Moreover, STING activation has been demonstrated to enhance cancer antigen presentation, contribute to the priming and activation of T cells, facilitate the trafficking and infiltration of T cells into tumors and promote the recognition and killing of cancer cells by T cells. In this review, we focus on STING and the cancer immune response, with particular attention to the roles of STING activation in the cancer-immunity cycle. Additionally, the negative effects of STING activation on the cancer immune response and non-immune roles of STING in cancer have also been discussed.

## Introduction

William Coley, the father of immunotherapy, began using *Streptococcus pyogenes* to treat patients with unresectable tumors in 1891 when chemotherapy and radiotherapy were not available [1]. Ultimately, Coley used a mixture of heat-inactivated *Streptococcus pyogenes* and *Serratia marcescens*, known as Coley’stoxin, to treat his cancer patients. For 40 years, Coley used his toxin to treat more than a thousand cancer patients, of which several hundred achieved near complete regression [2]. However, Coley did not know how toxins worked and did not figure out how inflammation treated tumors.

The discovery of phagocytosis by Mechnikov (a Nobel Prize winner) in 1883, led to the crucial understanding of the concept of innate immunity, and many great discoveries followed. Notably, innate immunity entered a new phase in the 1990s when Janeway proposed the concept of pathogen-associated molecular patterns (PAMPs) and pattern recognition receptors (PRRs) [3]. It is now widely accepted that innate immunity plays a critical role in the host defense against microbial infection by recognizing different microbial PAMPs via various PRRs in immune cells and initiating the production and secretion of interferons (IFNs) and cytokines, which then stimulate and activate the adaptive immune response [4].Toll-like receptors (TLRs) on the surface of immune cells are one of the well-known PRRs, and different TLRs recognize different PAMPs. For instance, TLR3, TLR7 and TLR9 recognize dsRNA, ssRNA and CpG DNA, whereas TLR1, TLR2, TLR4 and TLR5 recognize bacterial lipopeptides, peptidoglycan, lipopolysacchride (LPS) and flagellin, respectively (reviewed in ref. [5]). There are also some PPRs within the cytosol of immune cells, such as the NOD-like receptor (NLR), which recognizes bacterial cell-wall lipids and products from damaged host cells, and the RIG-like receptor (RLR), which recognizes viral RNA (reviewed in ref. [6, 7]).

Although it has been known that DNA can stimulate immune responses since as early as 1908 by Mechnikov [8], and numerous studies have demonstrated that the recognition of double-stranded DNA (dsDNA) by innate immune sensors contributes to the development of systemic lupus erythematosus (SLE), a well-known autoimmune disease [9], the dsDNA sensor within immune cells remained unidentified throughout the entire twentieth century. Before the identification of the dsDNA sensor, several groups made a great contribution to the field in 2008 and 2009 by identifying an ER protein, STING (stimulator of interferon genes), as a key component in DNA-mediated innate immunity [10–13]. In 2013, Dr. Chen’s group ultimately determined that cGAS is the direct cytosolic DNA sensor and that it activates innate immunity by activating type I IFN expression [14, 15].

Cytosolic DNA triggers the activation of cGAS-cGAMP-STING signaling. This signaling not only plays critical roles in the host defense against microbial infection, but also has been demonstrated to be involved in the antitumor immune response, and numerous studies have suggested that the activation of STING is a novel and promising strategy to treat cancer. In this review, we focus on STING and the cancer immune response and elaborate on the master roles of STING activation in regulating the cancer-immunity cycle.

## STING induces the production of type I IFN and activates the innate immune system

Whether caused by leakage from the nucleus or mitochondria or induced by viruses or bacteria, cytoplasmic DNA is a danger signal. Once in the cytoplasm, dsDNA or single-stranded DNA (ssDNA) is sensed by a DNA sensor protein, cGAS, in a sequence-independent but length-dependent manner; cGAS catalyzes the synthesis of 2′3’-cyclic GMP-AMP (2′3’-cGAMP) by using ATP and GTP as substrates [14, 15], and it acts as a second messenger to bind and activate STING.

**Fig. 1:**
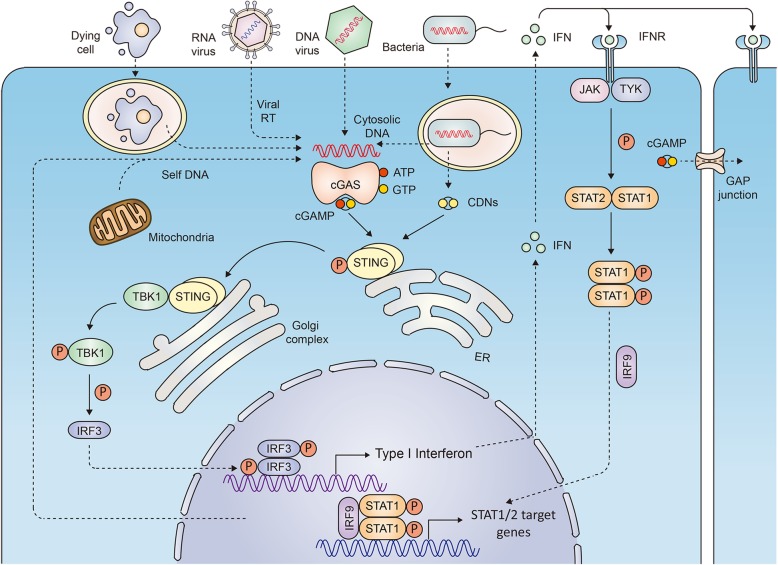
DNA-driven cGAS-cGAMP-STING signaling mediates innate immune response. The left cell exhibits the main components of cGAS-cGAMP-STING signaling pathway and IFN signaling pathway, and the right cell shows that IFN could activate neighbor cells in a paracrine manner and cGAMP could be transferred to neighbor cells through GAP junction

STING is a protein with four putative transmembrane domains and resides in the endoplasmic reticulum (ER) [12, 16], and it is widely expressed in both immune cells (including innate immune cells and adaptive immune cells) and non-immune cells. As a sensor of cyclic dinucleotides (CDNs), including both endogenous 2′3’-cGAMP catalyzed by cGAS in the presence of DNA and exogenous c-di-AMP, c-di-GMP or 3′3’-cGAMP from bacteria, STING binds to these small molecules, is activated, and translocates from the ER to the perinuclear area with the help of iRhom2, wherein STING activates the kinase TANK-binding kinase 1 (TBK1), which phosphorylates STING. Phosphorylated STING recruits interferon regulatory factor 3 (IRF3), which is phosphorylated by TBK1 and forms a homodimer to enter the nucleus and activates the transcription of type I IFNs and inflammatory cytokines and chemokines (Fig. 1) [17]. Notably, since cGAMP could be transferred via gap junction and through viral packaging, thus cGAMP may also activate STING in cells where cytoplasmic dsDNA is not available [18–20]. The modification and interaction with the components in this signaling pathway has been reviewed previously [17, 21, 22].

All type I IFNs (including well-documented IFN-α and IFN-β and less well-studied IFN-ε, IFN-κ, IFN-τ and IFN-ω) bind to heterodimer interferon receptors (IFNAR1 and IFNAR2). This results in the recruitment of Janus family kinase1 (Jak1) and tyrosine kinase 2 (Tyk2), and these, in turn phosphorylate and activate IFNAR1 and IFNAR2. The activation of IFNARs causes the recruitment and phosphorylation of effector proteins of the signal transducers and activators of transcription (STAT) family. Phosphorylated STAT1 and STAT2, together with IRF9, transfer to the nucleus, where they enhance the transcription of IFN target genes (reviewed in ref. [21, 23]), leading to the activation of both innate and adaptive immunity.

Numerous studies have shown that the expression levels of Type I IFNs and Type I IFN-induced genes in cancer cells positively correlate with T-cell infiltration in the tumor microenvironment [21]. Most importantly, IFNAR or STAT1 knockout mice fail to reject immunogenic tumors due to the less efficient induction of DC recruitment to tumors and the priming and expansion of CD8^+^ T cells in vivo [24–26]. Consistent with these studies, many previous studies also revealed that type I IFNs contribute to the control of tumors both in vivo and in vitro [27, 28]. These studies suggest that type I IFNs play central roles in the antitumor response. However, recent studies have suggested that type I IFNs may also impair anticancer immunity and even cause unexpected treatment failure for cancer. For example, IFN-β has been shown to induce the production of programmed cell death ligand 1 (PD-L1) and programmed cell death ligand 2 (PD-L2) in tumor cells [29, 30], which contributes to immune escape by cancer cells. Moreover, type I IFNs have been reported to be associated with resistance to radiotherapy and chemotherapy due to type I IFNs inducing DNA damage resistance in multiple cancer types [31, 32]. Additionally, type I IFNs have been revealed to contribute to unexpected autoimmune toxicity during cancer immunotherapy in the clinic [33]. Taken together, even though type I IFNs play central roles in anticancer immunity, immunotherapy directly based on type I IFNs may not be applicable in cancer treatment in the clinic.

It is currently believed that inducing the production of type I IFNs is one of the major mechanisms for STING signaling-mediated anticancer immunity. However, there is some evidence suggesting that STING also regulates anticancer immunity in a type I IFN-independent manner, which implies a broader application of STING (beyond IFNs) in cancer immunotherapy.

## Activation of STING is a promising strategy for the cancer immunotherapy

Recent studies have suggested that STING signaling is necessary for the anticancer immune response based on the following observations: on the one hand, STING knockout mice and IRF3 knockout mice show impaired spontaneous T-cell responses against tumors [34, 35]; on the other hand, STING agonists show a favorable effect in promoting the infiltration of T cells into the tumor microenvironment [36, 37]. Moreover, numerous studies using the STING agonists to treat cancers demonstrate that activation of STING is a promising strategy for the cancer immunotherapy.

Actually, before identified the STING signaling, a chemotherapeutic agent 5,6-dimethylxanthenone-4-acetic acid (DMXAA), first synthesized in 2002 as an antivascular agent, shows a promising anticancer effect, although the target molecules of DMXAA is unknown at the time [38]. Further studies show that the anticancer effect of DMXAA is associated with activation and infiltration of CD8^+^ T cells in murine models of several cancer types [39] and is dependent on type I INF production [40]. In 2012, DMXAA was finally shown to target STING and activate STING dependent type I INF induction [41]. As the first applied STING agonist in cancer immunotherapy, DMXAA showed promising antitumor activity in mice, but unfortunately, it failed in clinical trials because DMXAA does not preferentially bind to human STING [42, 43]. However, these researches strengthened the confidence of scientists to develop STING agonists to treat cancer. Nowadays, it has been demonstrated that STING activation is effective in anticancer in various cancer types, including hematological malignancies (such as acute myeloid leukemia and lymphoma) and solid tumors (such as lung cancer and melanoma). The roles of STING activation in different cancer types are summarized in Table 1.

**Table 1:** Roles of STING activation in cancer

Cancer types	Treatment information regarding STING activation	Biological roles of STING activation in Cancer	Reference
Acute meyloid leukemia	DMXAA, 450 μg, i.t.	Promote DC maturation and enhance CD8^+^ T cell responses via the induction of type I IFN	[44]
Breast cancer	Topotecan (TPT, an inhibitor of topoisomerase I), 20 mg/kg, i.p. Olaparib (PARP inhibitor), 50 mg/kg daily, i.p. c-di-GMP, 150 nM, 24 h and c-di-GMP, 0.01 nM, i.p. Mafosfamide, 10 μM	Mediate DC activation Increase CD8^+^ T cell infiltration Activate caspase-3 and kill tumor cell directly, improve CD8^+^ T cell responses and restrict MDSCs Activate IFN/STAT1 pathway and protect breast cancer cells from genotoxic agents	[45] [46] [47] [48]
Colorectal cancer	Gamma rays (6 Gy)	Induce type III IFN production after gamma-radiation by the activation of the cytosolic DNA sensors-STING-TBK1-IRF1 signaling pathway	[49]
	Radiation (40 Gy)	Promote type I IFN production and contribute to sensing irrated-tumor cells by DC Induce MDSC mobilization which mediates	[50]
	2′3’cGAMP, 10 μg / X-ray	radioresistance in mouse models	[51]
Glioma	c-di-GMP, 4 μg, i.t.	Enhance CD4^+^ and CD8^+^ T cell infiltration and migration into the brain via type I IFN signaling and other chemokines	[37]
Head and neck squamous cell carcinoma	Matrigel containing 25 μg cyclic-di-AMP (CDN)	Induce type I IFN in the host cells and promote CD8^+^T cell response	[52]
	cGAMP, 10 μg/ml, 24 h	Facilitate cetuximab mediated NK cell activation and DC maturation	[53]
	R, R-CDG, 20 μg, i.t.	Promote Th1 response and increase IFN-γ^+^CD8^+^, but upregulate PD-L1	[54]
	R, R-CDG, 15 μg, i.t.	Increase the production of type I and II IFN but also promote the expression of PD-1 pathway components	[55]
Lung cancer	PARP inhibitors	Promote infiltration and activation of lymphocytes in NSCLC and SCLC	[56, 57]
	DMXAA/2′3’-cGAMP, 20 μg/ml, 24 h	Re-educate M2 macrophages towards an M1 phenotype in murine NSCLC	[58]
	cGAMP, 10 μg, i.t.	Normalize tumor vasculature and augment the infiltration of CD8^+^ T cell in LLC tumor	[59]
Malignant lymphoma	3′3’-cGAMP, 20 μM, 4 h	Induce apoptosis of malignant B cells via IRE-1/XBP-1 pathway	[60]
Melanoma	Tumor derived DNA(B16), 1 h	Induce IFN-β production in APC and is indispensable for T cell activation and expansion	[35]
	2′3’ cGAMP, 200 nM, i.p.	Activate NK cell response	[61]
Nasopharyngeal carcinoma	EBV infection.	Restrict the secretion of GM-CSF and IL-6, thereby suppress the MDSC induction	[62]
Ovary cancer	2′3’-c-di-AM(PS) (Rp, Rp), 4 mg/kg, i.p.	Increase the infiltration of activated CD8^+^ T cell into tumors	[63]
Pancreatic cancer	DMXAA, 300/450 μg, i.t.	Promote trafficking and activation of tumor-killing T cells, decrease the infiltration of Treg, and reprogram immune-suppressive macrophages	[64]
Prostate Cancer	Cytosolic DNA generated by endonuclease MUS81	Induce type I IFN expression and mobilize phagocytes and promote T cell responses	[65]
	c-di-GMP, 25 μg, i.t.	Provoke abscopal immunity	[66]
Tongue squamous cell carcinoma	HPV infection.	Enhance Treg infiltration through upregulation of CCL22 expression in HPV+ tongue squamous cells	[67]

*i.t.* Intratumoral injection

*i.p.* Intraperitoneal injection

*R, R-CDG* Synthetic CDN RP, RP dithio c-di-GMP

*NSCLC* Non-small cell lung cancer

*SCLC* Small cell lung cancer

*EBV* Epstein-Barr virus

*HPV* Human papilloma virus

In addition to DMXAA, there are other types of STING agonists have been developed, and the anticancer effect of those agents has been tested or under evaluated in clinic. CDNs, such as cGAMP and c-di-AMP, synthesized or acquired from microbes, represent the natural agents to bind and activate STING. However, these STING agonists are nonpenetrating [68], thus they must be delivered into cells via vectors, such as liposomes or nanoparticles [69]. Currently, some groups are developing novel CDN derivatives to perform clinical trials [70, 71]. In contrast, a very recent study reported a novel STING agonist, diABZIs, which is a small molecule developed based on amidobenzimidazole (ABZI) symmetry rather than CDNs that showed strong and systemic antitumor activity in a mouse colon cancer model [71]. The clinical studies using the STING agonists in different cancer types are summarized in Table 2.

**Table 2:** Clinical trials of STING agonists in cancer therapy

Identifier	STING agonist	Sponsor/ collaborator	Study tittle	Cancer types	Status
NCT00863733	DMXAA (ASA 404)	Cancer Research UK and Cancer Society Auckland	Study of DMXAA (Now Known as ASA404) in Solid Tumors	Solid Tumors	Completed
NCT00856336	DMXAA (ASA 404)	Antisoma Research	Phase I Safety Study of DMXAA in Refractory Tumors	Refractory Tumors	Completed
NCT00832494	DMXAA (ASA 404)	Antisoma Research	Phase II Study of DMXAA (ASA404) in Combination with Chemotherapy in Patients with Advanced Non-Small Cell Lung Cancer	Non-Small Cell Lung Cancer	Completed
NCT01299415	DMXAA (Vadimezan™)	Novartis	Safety and Pharmacokinetics of ASA404 When Given Together with Fluvoxamine, a Selective Serotonin Receptor Reuptake Inhibitor and CYP1A2 Inhibitor	Solid Tumors	Terminated
NCT01290380	DMXAA (ASA 404)	Novartis	A Study to Evaluate the Effects of ASA404 Alone or in Combination with Taxane-based Chemotherapies on the Pharmacokinetics of Drugs in Patients with Advanced Solid Tumor Malignancies	Solid Tumor Malignancies	Terminated
NCT01299701	DMXAA (ASA 404)	Novartis	A Single Center Study to Characterize the Absorption, Distribution, Metabolism and Excretion (ADME) of ASA404 After a Single Infusion in Patients with Solid Tumors	Advanced Solid Tumors	Terminated
NCT01278758	DMXAA (ASA 404)	Novartis	A Dose-escalation Pharmacokinetic Study of Intravenous ASA404 in Adult Advanced Cancer Patients with Impaired Renal Function and Patients with Normal Renal Function	Metastatic Cancer	Terminated
NCT01285453	DMXAA (ASA 404)	Novartis	Safety and Tolerability of ASA404 Administered in Combination with Docetaxel in Japanese Patients with Solid Tumors	Advanced or Recurrent Solid Tumors	Completed
NCT01278849	DMXAA (ASA 404)	Novartis	An Open-label, Dose Escalation Study to Assess the Pharmacokinetics of ASA404 in Adult Cancer Patients with Impaired Hepatic Function	Histologically-proven and Radiologically-confirmed Solid Tumors	Terminated
NCT00674102	DMXAA (ASA 404)	Novartis	An Open-label, Phase I Trial of Intravenous ASA404 Administered in Combination with Paclitaxel and Carboplatin in Japanese Patients with Non-Small Cell Lung Cancer	Non-small Cell Lung Cancer	Completed
NCT01071928	DMXAA (ASA 404)	Hoosier Cancer Research Network And Novartis	Second-Line Docetaxel + ASA404 for Advanced Urothelial Carcinoma	Urothelial Carcinoma	Withdrawn
NCT00856336	DMXAA (ASA 404)	Antisoma Research	Phase I Safety Study of DMXAA in Refractory Tumors	Refractory Tumors	Completed
NCT00832494	DMXAA (ASA 404)	Antisoma Research	Phase II Study of DMXAA (ASA404) in Combination with Chemotherapy in Patients with Advanced Non-Small Cell Lung Cancer	Non-Small Cell Lung Cancer	Completed
NCT01240642	DMXAA (ASA 404)	Novartis	An Open-label, Dose Escalation Multi-Center Study in Patients with Advanced Cancer to Determine the Infusion Rate Effect of ASA 404 With Paclitaxel Plus Carboplatin Regimen or Docetaxel on the Pharmacokietics of Free and Total ASA404	Metastatic Cancer with Impaired Renal Function Metastatic Cancer with Normal Renal Function	Terminated
NCT00111618	DMXAA (ASA 404)	Antisoma Research	Study of AS1404 With Docetaxel in Patients with Hormone Refractory Metastatic Prostate Cancer	Prostate Cancer	Completed
NCT01057342	DMXAA (ASA 404)	Swiss Group for Clinical Cancer Research	Paclitaxel, Carboplatin, and Dimethylxanthenone Acetic Acid in Treating Patients with Extensive-Stage Small Cell Lung Cancer	Lung Cancer	Completed
NCT01031212	DMXAA (ASA 404)	University of California, San Francisco and Novartis	ASA404 in Combination with Carboplatin/Paclitaxel/Cetuximab in Treating Patients with Refractory Solid Tumors	Tumors	Withdrawn
NCT00662597	DMXAA (ASA 404)	Novartis	ASA404 or Placebo in Combination with Paclitaxel and Carboplatin as First-Line Treatment for Stage IIIb/IV Non-Small Cell Lung Cancer	Non-Small Cell Lung Cancer	Terminated
NCT03937141	MIW815 (ADU-S100)	Aduro Biotech, Inc	Efficacy and Safety Trial of ADU-S100 and Anti-PD1 in Head and Neck Cancer	Metastatic head and neck cancer Recurrent head and neck cancer	Recruiting Phase 2
NCT02675439	MIW815 (ADU-S100)	Aduro Biotech, Inc. and Novartis	Safety and Efficacy of MIW815 (ADU-S100) +/− Ipilimumab in Patients with Advanced/Metastatic Solid Tumors or Lymphomas	Solid tumors Lymphomas	Recruiting Phase 1
NCT03172936	MIW815 (ADU-S100)	Novartis	Study of the Safety and Efficacy of MIW815 With PDR001 to Patients with Advanced/Metastatic Solid Tumors or Lymphomas	Solid tumors Lymphomas	Recruiting Phase 1
NCT03010176	MK-1454	Merck Sharp and Dohme Corp.	Study of MK-1454 Alone or in Combination with Pembrolizumab in Participants with Advanced/Metastatic Solid Tumors or Lymphomas	Solid tumors Lymphomas	Recruiting Phase 1

## STING signaling regulates the cancer-immunity cycle

**Fig. 2:**
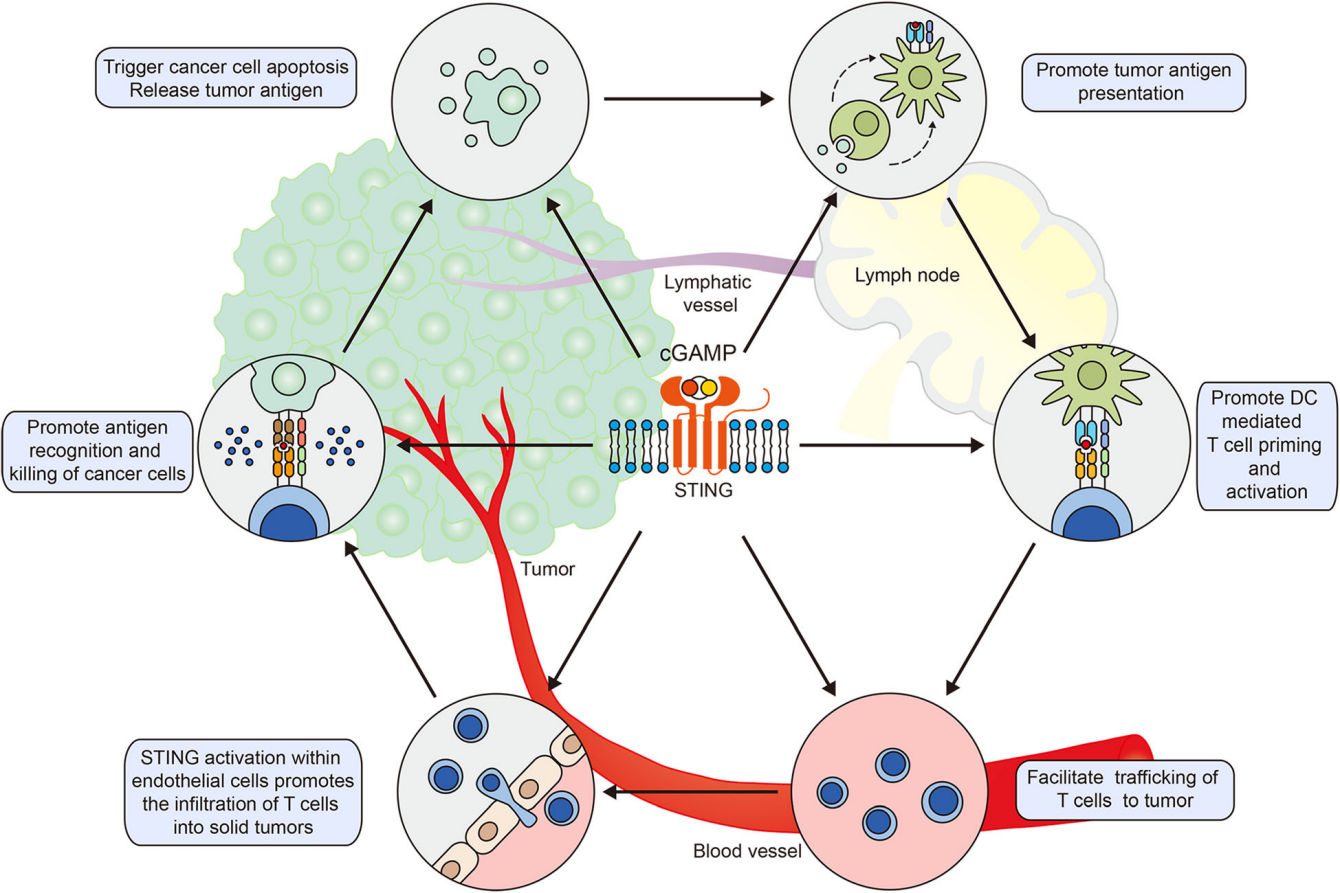
Activation of STING positively regulates each step of cancer-immunity cycle

Cancer cell death results in the exposure of cancer antigens; antigen-presenting cells (APCs), typically referred to as dendritic cells (DCs), capture the antigens and present them to T cells, and induce the activation of effector T cells. Next, effector T cells reach the tumor site and infiltrate tumors, where cytotoxic T lymphocytes (CTLs) identify and kill cancer cells. In turn, dead cancer cells release more antigens, which participate in the process above. This cyclic process is defined as the cancer-immune cycle [72]. The cancer-immunity cycle has become a research hotspot in recent years and provides a theoretical basis for tumor immunotherapy. There are a series of stimulatory and inhibitory factors involved in this cyclic process [72]. STING, as a stimulator of type I IFN production, has been demonstrated by an increasing number of studies to act as a master regulator and mediator in each step of the cancer-immunity cycle (Fig. 2).

### STING facilitates the release of cancer cell antigens

Tumor cells are main reason of producing cancer antigens, arise due to genome instability and high exposure to few oncogenes. However, these antigens cannot clearly seen, due to mutation or deletion of the MHC-coding genes in the cancer cells [73], which makes tumor to deceive the immune system. Therefore, APCs has ability to consume the proteins and even mRNAs coding for cancer antigens released by inactive tumor cells, which makes them to appear on the surface of APCs. Thus, this release starts the development of the cancer-immune cycle.

Recent studies have found that the activation of STING can directly trigger cancer cell death. Tang et al. reported that the STING agonist 3′3’-cGAMP is cytotoxic to malignant B cells and induces apoptosis in vitro and in vivo [60]. Mechanistically, they found that 3′3-cGAMP binds to STING and causes the phosphorylation and activation of STING in mouse embryonic fibroblasts. However, this agonist promotes the degradation of STING protein upon binding to it, and this process requires STING to interact with the ER stress sensor IRE-1. Unlike mouse embryonic fibroblasts, the 3′3’-cGAMP-STING interaction causes STING to aggregate in malignant B cells and leads to rapid apoptosis of these cells [60]. In addition to this, researches showed that the infection with human T-cell leukemia virus (HTLV-1) in monocytes, which become a reason of reversing transcription intermediates of HTLV-1, in order to collaborate with STING within the cytoplasm. This causes the production of an IRF3-Bax complex, which results in apoptosis of HTLV-1-infected monocytes [74].

Recently, it has been demonstrated that major histocompatibility complex class II (MHC-II) causes apoptosis of hematopoietic malignant cells [75, 76]. It has been revealed that STING protein is associated with MHC-II and mediates apoptosis of B lymphoma cells. Mechanistically, MHC-II aggregation results in tyrosine phosphorylation of STING, which triggers the activation of the extracellular signal-regulated kinase (ERK) signaling pathway and this process is necessary for MHC-II-mediated cell death signaling in a murine B lymphoma cell line [16]. Although MHC-II molecules have been reported to express in various cancer types [77–79], it is still not clear about the roles of the interaction of STING and MHC-II in inducing apoptosis of non-hematopoietic malignant cells currently. These studies suggest that STING activation and/or overexpression may trigger cell apoptosis and cause the release of tumor antigens in certain cancer types.

### Activation of STING signaling is necessary for cancer antigen presentation

It has been demonstrated that radiation and chemotherapeutic agents induce antitumor immune responses depending on type I IFN when used to directly attack cells, and that STING is essential for such radiation-induced immune responses [50, 80]. Emerging evidence also indicates that dying cells can release endogenous adjuvant and facilitate activation of APCs [81]. When suffering nonphysiological damage, tumor cells release numerous danger-associated molecular patterns (DAMPs), which can trigger host immune responses [82]. Tumor cell-derived DNA is one of the most important DAMPs. DNA released from dead tumor cells can be found within the cytosol of intratumoral DCs [34]. Tumor-derived DNAs can be recognized by cytoplasmic DNA receptors in dendritic cells, macrophages, and other APCs and activate the cGAS-STING pathway to induce the expression of type I IFN [50].

DCs are the most potent professional APCs, and DC activation and antigen presentation are regulated by multiple factors, and type I IFN plays a particularly crucial role in the regulation of DCs. As early as 1998, T. Luft et al. demonstrated that type I IFN enhances the terminal differentiation of DCs [83]. Since then, R.L. Paquette [84] and L.G. Radvanyi [85] have found that type I IFN also facilitates the maturation of DCs. Recent studies have found that in addition to promoting DC maturation by inducing the expression of type I IFN, cGAMP or other STING agonists can directly activate DCs in vitro, and enhance presentation of tumor-associated antigens to CD8^+^ T cells [86, 87]. Furthermore, activation of STING signaling in DCs can induce additional protein expression to promote cross-presentation and T-cell activation [88]. Therefore, these studies suggest that, in order to generate adaptive antitumor immunity, STING must be activated by tumor-derived DNA or cGAMP for IFN expression and DC-mediated cross-priming.

### STING signaling is responsible for the priming and activation of T cells

The priming and activation of T cells involve multiple signals, including T cell receptor (TCR) recognition and interaction with costimulatory molecules. In addition, cytokines play important roles in T-cell activation. It has been revealed that spontaneous T-cell priming and activation occur in the tumor microenvironment of some solid tumors [89]. Current research suggests that spontaneous tumor antigen-specific T-cell priming appears to be dependent on DC and type I IFN production in host cells [26].

Recently, Seng-Ryong Woo et al. reported that spontaneous T-cell priming was severely debilitated in STING-deficient and IRF3-deficient mice [35], and Olivier Demaria et al. also observed the same phenomenon in STING knockout mice compared with WT mice [36], which suggests that STING signaling may be necessary for the expansion of T cells. In addition, Olivier Demaria et al. [36] and Juan Fu et al. [90] both reported that mice with B16 melanoma treated with cGAMP showed an increase in CD8^+^ T-cell infiltration in the tumor microenvironment. These results imply that STING activation could facilitate T-cell priming and activation in the tumor microenvironment. However, these studies did not elaborate the detail mechanisms of STING signaling regulating this process. Since DCs and type I IFNs play critical roles in the priming and activation of T cells, and it has been revealed that tumor-derived DNA activates DCs and induces production of type I IFN in the tumor microenvironment [34], thus activation of STING signaling in DCs plays important and even exclusive roles in the spontaneous T cell responses against tumors. When it comes to applying STING agonists to stimulate T cell responses against tumors, T cells could be directly activated by STING agonists [91, 92] and indirectly activated by type I IFN produced by STING activated DCs.

### Activation of STING pathway promotes the trafficking and infiltration of T cells to tumors

Before recognizing and killing cancer cells, CTLs must traffic to and infiltrate the tumor tissue. Chemokines play essential roles in regulating the development, priming, functions, homing and retention of T cells (reviewed in ref. [93, 94]). Previous studies demonstrated that the infiltration of CD8^+^ T cells in the tumor microenvironment is associated with C-X-C motif chemokine ligand 9 (CXCL9), C-C motif chemokine 5 (CCL5) and C-X-C motif chemokine ligand 10 (CXCL10) [95], and the expression of CXCL9 and CXCL10 could be induced in response to type I IFN production by APCs [96], which suggests that APCs play important roles in the trafficking and infiltration of CD8^+^ T cells. Recently, L Corrales et al. reported that elevated expression of CXCL9 and CXCL10 in DCs is associated with the activation of the STING pathway and contributes to trafficking and infiltration of CD8^+^ T cells in a xenograft animal model [97]. In addition to DCs, some other immune cells have also been found to be involved in STING-mediated T-cell trafficking. For example, Ohkuri T et al. observed macrophage aggregation after intratumoral injection of cGAMP in mice; however, no aggregation was observed in STING knockout mice. After depletion of mouse macrophages, the antitumor effect induced by cGAMP disappeared, and the mechanistic analysis revealed that STING-induced migrating tumor macrophages express high levels of T-cell-recruiting chemokines, such as CXCL10 and C-X-C motif chemokine ligand 11 (CXCL11), which then contribute to CD8^+^ T-cell trafficking to the tumor site [98]. In another study, it has been revealed that intratumoral injection of STING agonist (c-di-GMP) activated STING/type I IFN signaling in the CD11b^+^ brain-infiltrating leukocytes (instead of CD11c^+^ DCs), in which CXCL10 and CCL5 expression was increased and then contributed to the migration of CD8^+^ T cells into the glioma [37]. These results show that activation of the STING pathway in APCs and other immune cells can induce the expression of cytokines and thereby promote T-cell trafficking.

Other than immune cells, recent researches showed that the STING activation within endothelial cells causes the infiltration of T cells into solid tumors. Demaria and colleagues found that spontaneous infiltration of CD8^+^ T cells in an engrafted melanoma is significantly reduced in STING knockout mice compared with WT mice. Furthermore, they demonstrated that intratumoral injection of cGAMP promotes the infiltration of CD8^+^ T cells into engrafted melanoma [36]. Mechanistically, they revealed that STING-induced IFN-β contributes to the infiltration of CD8^+^ T cells because the blockage of IFN signaling by anti-IFNAR antibodies or IFNAR ablation completely abolished CD8^+^ T-cell infiltration [36]. By detecting the expression of intracellular IFN-β within tumor-cell-derived single cells, the authors revealed that IFN-β-producing cells in the tumor express low levels of CD45 (a general marker of hematopoietic cells) but high levels of CD31 and vascular endothelial growth factor receptor 2 (VEGFR-2) (the specific marker of endothelial cells), suggesting that activation of STING pathway by exogenous STING agonists in endothelial cells, instead of DC cells or other immune cells, facilitate the infiltration of CD8^+^ T cells into the tumor microenvironment [36]. Consistently, another study also found that STING expression in endothelial cells is positively correlated with the infiltration level of CD8^+^ T cells and prolonged survival in several human cancer types (eg. colon and breast cancer) by using immunohistochemistry staining [59]. However, authors revealed that non-hematopoietic cells play important roles in the infiltration of CD8^+^ T cells into tumor microenvironment by employing bone morrow chimeric mice models, they did not show the direct evidence to illustrate the accurate roles of STING activation in endothelial cells in the process of T cell infiltration [59]. These results revealed an unexpected role of endothelial cells within the tumor microenvironment in cancer immunity, and suggested that STING activation in endothelial cells is necessary for the infiltration of CTLs.

Adhesion to endothelial cells is a necessary step for the infiltration of T cells into the tumor microenvironment. Current studies have demonstrated that vascular endothelial growth factor (VEGF) and other cytokines secreted by cancer cells inhibit the expression of molecules on endothelial cells that mediate the adhesion of T cells or induce the expression of molecules that trigger cell death of effecter T cells (reviewed in ref. [99, 100]). Moreover, the depletion of CD8^+^ T cells has been shown to abrogate the therapeutic efficacy of VEGF inhibition by using an anti-VEGFR antibody in a certain cancer model [101]. Thus, inhibition of VEGF signaling promotes the infiltration of T cells into the tumor microenvironment. Consistent with these studies, Hannah and colleagues demonstrated that STING agonists (10 μg of cGAMP or 25 μg ofRR-CDA) treatment combined with VEGFR2 blockade (DC101) enhanced the infiltration of CD8^+^ T cells in the tumor microenvironment and induces complete tumor regression [59], this exciting result suggests that simultaneously targeting STING and VEGF signaling represents a promising strategy for cancer therapy. However, it must be aware that combined using immunotherapy and anti-angiogenic therapy targeting VEGF or VEGFR may be not effective in certain conditions, because it has been indicated that VEGF inhibition is not beneficial in some human solid tumor types (NSABP-C-08; clinicaltrials.gov: NCT00096278) and even results in progression in certain cancer types (reviewed in [102, 103]). These unexpected phenomenons may be partially explained by that the blockade of VEGF may inhibit the infiltration of T cells by suppressing the proliferation of endothelial cells within tumors in some conditions because appropriate level of VEGF is necessary for maintaining the number of endothelial cells.

Although multiple studies have found that injection of a STING agonist in a tumor-bearing mouse model enhanced the infiltration of T cells into the tumor microenvironment [37, 90], and several types of cells, such as DCs, macrophages and endothelial cells, have been identified to help infiltration of T cells into tumor microenvironment in responding to activation of STING pathway by exogenous STING agonists in different models, the direct effect of STING activation within T cells on their trafficking and infiltration is not evaluated currently. An in vitro study showed that exogenous STING agonist DMXAA activates STING signaling, and then induces type I IFN production and IFN-stimulated gene expression [91, 92], thus the STING activated CTLs by exogenous STING agonists may mirror the response of innate cells and induce more CTLs to migrate and infiltrate into tumor microenvironment. However, further studies are needed to detect this hypothesis.

### STING activation is necessary for the recognition and killing of cancer cells by T cells

Antigen binding by MHC followed by recognition and interaction with the TCR is a critical step for T-cell recognition of cancer cells [104]. After recognizing tumor cells, activated CTLs can release cytokines, such as IFN-γ and other factors, to mediate tumor cell death [105].

Numerous studies have reported that STING activation promotes the antitumor effect of CD8^+^ T cells. It has been reported that antigen-specific CD8^+^ T-cell responses were diminished in STING-deficient in a murine radiation-mediated antitumor immunity model [50]. Consistently, another study also revealed that the CD8^+^ T-cell response to tumor-associated antigens was diminished in both STING-deficient and IRF3-deficient mice [35]; these data suggest that host-cell STING and IRF3 are required for spontaneous CD8^+^ T-cell activity against immunogenic tumors. Furthermore, Ohkuri T et al. found that STING-deficient mice had fewer IFN-γ-producing CD8^+^ T cells but increased infiltration of immune-suppressing cells, such as CD11b^+^Gr-1^+^ immature myeloid suppressor cells and CD25^+^Foxp3^+^ regulatory T (Treg) cells, in the tumor microenvironment [37], whereas STING agonist CDN treatment promoted cross-presentation and helped T cells recognize tumor cells [106]. These data suggest significant contributions of STING to T-cell-mediated antitumor immunity via enhancement of type I IFN signaling in the tumor microenvironment.

## STING activation negatively regulates cancer immunity

Current studies show that STING activation facilitates the antitumor immune response in most conditions; however, emerging studies also suggest a potential inhibitory effect of STING activation on antitumor immune responses.

Although numerous studies have suggested that STING activation by exogenous cGAMP facilitates the priming and activation of T cells, two recent independent critical studies showed that STING activation in T cells prevents their proliferation and even promotes their death [91, 92]. The proliferation of T lymphocytes with constitutively active STING mutations was found to be impaired; the impairment was dependent on nuclear factor κB (NF-κB) instead of TBK1 and IRF3, due to mitotic errors resulting from STING relocalization to the Golgi apparatus after activation [91]. In another study, it was shown that STING agonist DMXAA activates the cell stress pathways within T cells and finally induces cell death of T cells [92]. These two studies suggest that STING activation in T cells could directly impair the adaptive immune system.

Additionally, it has been suggested that activation of STING signaling also activates immune suppressive cells in certain conditions. We evaluated the relationship between STING expression and the infiltration of 28 types of immune cells in 17 human malignant tumor types based on the TCGA data set and showed that the STING pan-cancer expression level is positively correlated with the infiltration of almost all types of immune cells, including both antitumor immune cells, such as DCs and CTLs, and immune-suppressing cells, such as myeloid-derived suppressor cells (MDSCs) and Tregs [107]. Our unexpected finding is consistent with several previous studies. A study in HPV+ tongue squamous cell carcinoma (TSCC) indicated that activated STING has no impact on cancer cell viability but promotes the induction of immunosuppressive cytokines, such as IL-10, which facilitated the infiltration of Tregs [67], whereas enriched Tregs can express IL-10 to inhibit the proliferation and activity of antigen-specific T cells [104]. In another study, Lemos and colleagues found that STING signaling contributes to the growth of Lewis lung carcinoma (LCC) by promoting the infiltration of MDSCs while decreasing the infiltration of CD8^+^ T cells in the tumor microenvironment [108]. Mechanistically, they revealed that the indoleamine 2,3-dioxygenase (IDO) activity is elevated significantly in LCC tumor microenvironment from WT mice compared with STING knockout or IFNAR knockout mice. Moreover, the effect of STING promoting tumor growth in LCC model is attenuated either knocking out IDO gene or following treatment with IDO inhibitors [108], suggesting that induction of IDO plays central roles in STING activation mediated tumor growth.

IDO is considered an enzyme, which accelerates the transformation of tryptophan into kynurenine. This is incurred due to innate immune response. It also plays a counter-regulatory role in the inflammation and activation of T cells [109]. IDO is also responsible to vanquish the effector’s T cells by doing metabolic depletion of tryptophan and formation of kynurenine. The depletion tryptophan restricts the escalation of both CD8^+^ and CD4^+^ T cells, from the local microenvironment, through blocking the ribosomal translation, incurred due to amino acid withdraw (this process is controlled by molecular stress-response pathways, reviewed in ref. [110–112]). On the other hand, kynurenine boost the differentiation of Foxp3^+^ Tregs, along with negatively regulating the dendritic cell immunogenicity via binding and activating a ligand-activated transcription factor AhR (aryl hydrocarbon receptor) [113, 114].

Enhanced IDO activity is commonly observed in the tumor microenvironment and is believed to be associated with cancer immune evasion (reviewed in ref. [115]). In addition to a recent study directly demonstrated that STING activation contributes to tumor growth [108], there are also many previous studies found that systemic treatment with DNA-containing nanoparticles stimulates IDO activity in many mouse tissues due to STING activation in innate immune cells [116], which activates Tregs and suppresses the T cell responses [117].

Notably, although the impact of STING agonists on the activation of IDO in the immune system or tumor microenvironment has not yet been evaluated, this potential effect on IDO activation must be investigated before applying STING agonists to treat cancer, whereas combined using IDO inhibitors may enhance the immunotherapeutic effect of STING agonists.

**Fig. 3:**
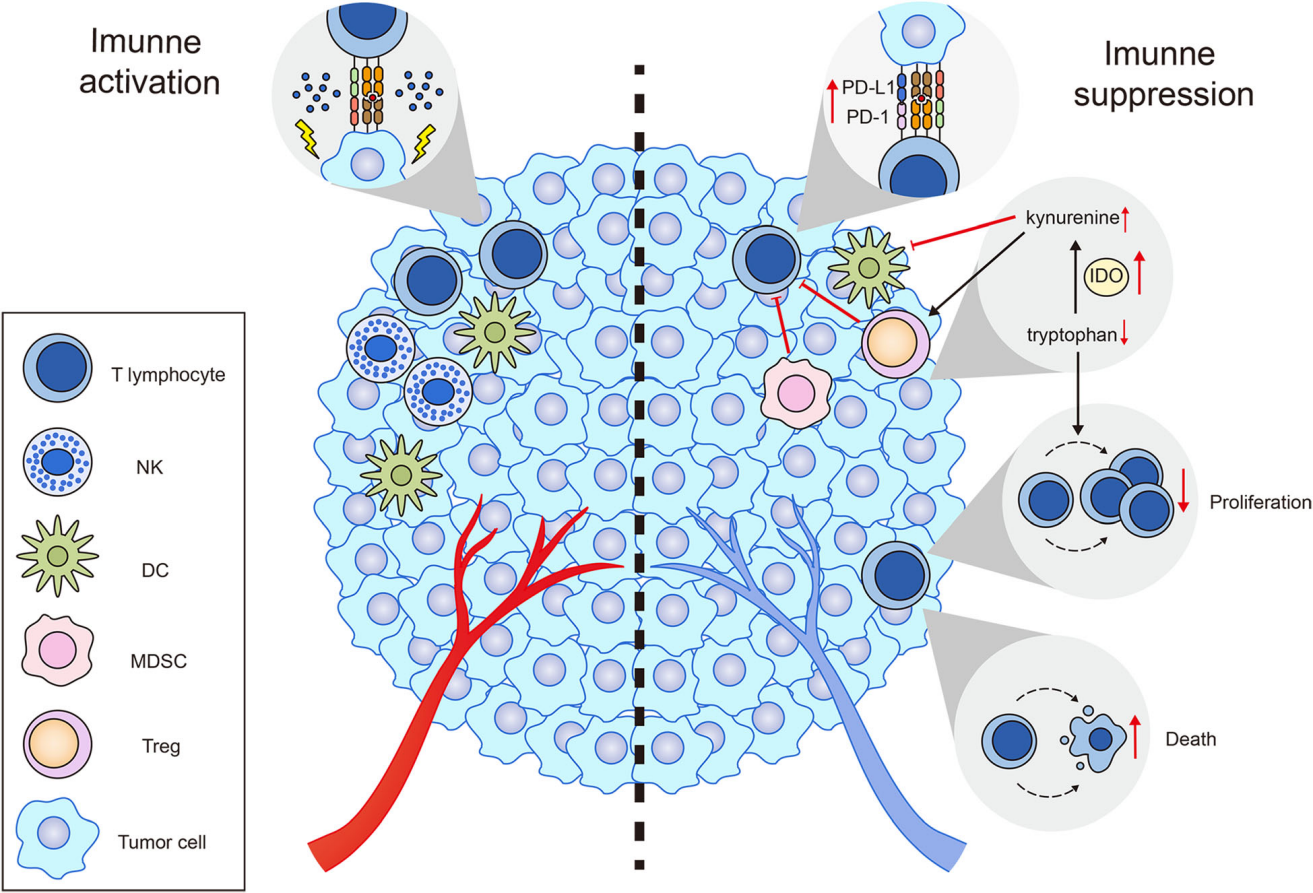
The positive and negative roles of STING activation in antitumor immune response. On the one hand, STING facilitates antitumor immune response through promoting the infiltration of effector cells and eradication of tumor cells. On the other hand, constant STING activation may hamper immune response by inducing the infiltration of immune suppressive cells, such as Treg and MDSC, and upregulating the expression of PD-L1 on tumor cells and PD-1 on T cells. Moreover, STING activation is associated with the enhanced activity of IDO, an enzyme catalyzing the transformation of tryptophan into kynurenine. Diminished tryptophan restricts the proliferation of T cells whereas elevated kynurenine promotes differentiation of Tregs but hampers antigen presenting ability of DCs. Additionally, aberrant STING activation also directly inhibits T cell proliferation and even promotes apoptosis of lymphocytes

In addition to Tregs, programmed cell death1 (PD-1) and other immune checkpoint molecules are also involved in inhibiting Tcell-mediated immunity [118, 119]. Recent studies showed that the activation of STING by c-di-GMP in infiltrated CD8^+^ T cells results in increased expression of PD-1 pathway components in multiple murine cancer types, including colon, tongue squamous carcinoma, pancreatic carcinoma and head and neck squamous cell carcinoma models [55, 90]. However, combined with the PD-1 pathway blockade, the increased expression of PD-1 is beneficial to the antitumor effect of STING agonisits [55, 90]. Together, these studies suggest that apart from playing positive roles in anticancer immune response, STING may hamper the antitumor immune response after it is inappropriately activated (Fig. 3).

## Non-immune functions of STING

In addition to regulating anticancer immunity, the non-immune functions of STING are emerging.

Firstly, STING activation results in cell apoptosis. For instance, STING agonists cause apoptosis of certain immune cells, including B cells and even T cells, in vitro and in vivo [60, 91, 92]. In addition to immune cells, activation of STING signaling also induces hepatocyte apoptosis in early alcoholic liver disease. Ethanol causes ER stress and triggers phosphorylation and activation of IRF3 by interacting with STING, activated IRF3 associates with Bax and induces apoptosis of hepatocytes, whereas deficiency of STING prevents hepatocyte apoptosis [120].

Secondly, STING mediates autophagy. For example, by sensing bacterial or viral PAMPs, STING signaling is activated and triggers ER stress; subsequently, STING localizes to autophagosomes from the ER, which provides a homeostatic mechanism to balance immunity and survival after infection [121, 122]. Liu et al. reported that STING directly interacts with LC3 and induces autophagy; however, cGAMP binding to STING activates the immune response, but the complex fails to interact with LC3 and reduces autophagy [123].

A very recent study confirmed that STING translocates to the endoplasmic reticulum-Golgi intermediate compartment (ERGIC) upon binding cGAMP, and this STING-containing ERGIC, which is a membrane source for the autophagosome biogenesis, through autophagy-related protein 5 (ATG5) and WD repeat domain phosphoinositide-interacting protein 2 (WIPI2) dependent pathway [124]. No doubt, the STING molecule regulates autophagy process, but the crosstalk between autophagy and immune response upon cGAMP binding STING needs further experimentation to explore.

Thirdly, STING also regulates cell proliferation by regulating the cell cycle. Ranoa and colleagues found that STING knockout in human and murine cancer cells lead to increased proliferation compared with wild-type controls. Mechanistically, they revealed that STING deficiency results in activation of cyclin-dependent kinase 1 (CDK1) and facilitates onset of S and M phase of the cell cycle in P53-activated P21 dependent manner [125]. This study implies that STING not only regulates cell death or survival, but also affects cell proliferation.

Fourthly, STING activation contributes to normalization of the tumor vasculatures. Chemotherapeutic agent DMXAA was firstly designed as an antivascular agent before it was identified to target STING [38], this widely used STING agonist shows rapid and strong antivascular activity in tumors but not in normal tissues, and it is effective to control tumor growth by regulating the vasculatures in various murine cancer models (reviewed in ref. [126]). Consistently, a very recent study also reported that intratumoral injection of other STING agonists (cGAMP or RR-CDA) normalizes the tumor vasculatures in spontaneous or implanted cancers, but this phenomenon is not observed in the STING deficient mice, which implies that STING activation is necessary for the normalization of the tumor vasculatures [59]. Mechanistically, they revealed that endothelial STING activation upregulates vascular stabilizing genes, such as Angpt1, Pdgfrb, and Col4a, in a type I IFN signaling dependent manner [59]. Notably, when combined with VEGFR2 blockade, STING agonists cause the complete regression of immunotherapy-resistant tumors [59]. These studies suggest that STING signaling in the tumor microenvironment regulates angiogenesis.

Finally, several studies found that activation of STING facilities cancer metastasis. It has been reported that the metastatic cancer cells transfer cGAMP to the astrocyte through carcinoma-astrocyte gap junctions, in which cGAMP activates STING pathway and induces production of inflammatory cytokines, these factors activate STAT1 and NF-κB pathway in cancer cells and thereby facilitate the survival and growth of metastatic cancer cells [127]. A similar study was done, which found that chromosomal instability also become a reason of accumulation of micronuclei in the cytoplasm of cancer cells, which results in activation of the STING pathway and downstream the NF-κB signaling, thereby promoting cancer metastasis [128].

Taking these considerations together, it is necessary to evaluate the non-immune functions of STING before using STING agonists to treat cancer in the clinic.

## Concluding remarks and perspectives

Since STING plays a critical role in innate immunity, the potential application of STING regulation in infectious diseases, autoimmune diseases and cancer has attracted great interests. In this review, we focused on the roles of STING in cancer immunity by elaborating on its effect at each step of the cancer-immunity cycle. Conclusively, STING is a potent regulator of cancer immunity functioning at each step of the cancer-immunity cycle, and thus activation of STING represents a promising strategy for cancer immunotherapy by developing safe and efficient STING agonists. However, accompanied with the STING-mediated activation of antitumor immune responses, potential immune inhibitory effects of STING are emerging and nonnegligible. In addition to this, it has been observed that STING activation also contributes in cancer initiation and progression, by activating cancer associated inflammation, when it induces type I IFN responses. Thus, it must be thoroughly evaluated, before the STING agonists are used to stimulate the anticancer immune response, and combined using immune checkpoint blockade therapy, such as IDO inhibitor, anti-PD-L1 and anti-PD-1 antibodies, may increase the therapeutic effects of STING agonists in the clinic.

## Data Availability

Not applicable.

## Abbreviations

ABZI: Amidobenzimidazole
AhR: Aryl hydrocarbon receptor
APC: Antigen-presenting cells
ATG5: Autophagy-related protein 5
CCL5: C-C motif chemokine 5
CDK1: Cyclin-dependent kinase 1
CDN: Cyclic dinucleotide
cGAMP: Cyclic GMP-AMP
CTL: Cytotoxic T lymphocytes
CXCL10: C-X-C motif chemokine ligand 10
CXCL11: C-X-C motif chemokine ligand 11
CXCL9: C-X-C motif chemokine ligand 9
DAMP: Danger-associated molecular pattern
DC: Dendritic cell
DMXAA: 5,6-dimethylxanthenone-4-acetic acid
dsDNA: Double-strand DNA
EBV: Epstein-Barr virus
ER: Endoplasmic reticulum
ERGIC: Endoplasmic reticulum-Golgi intermediate compartment
ERK: Extracellular signal-regulated kinase
HPV: Human papilloma virus
HTLV-1: Human T-cell leukemia virus
IDO: Indoleamine 2,3-dioxygenase
IFN: Interferon
IRF3: Interferon regulatory factor 3
Jak1: Janus family kinase1
LCC: Lewis lung carcinoma
LPS: Lipopolysacchride
MDSCs: Myeloid-derived suppressor cell
MHC-II: Major histocompatibility complex class II
NF-κB: Nuclear factor κB
NLR: NOD-like receptor
NSCLC: Non-small cell lung cancer
PAMP: Pathogen-associated molecular pattern
PD-1: Programmed cell death 1
PD-L1: Programmed cell death ligand 1
PD-L2: Programmed cell death ligand 2
PRR: Pattern recognition receptor
R, R-CDA: RP, RP dithio c-di-AMP
R, R-CDG: RP, RP dithio c-di-GMP
RLR: RIG-like receptor
SCLC: Small cell lung cancer
SLE: Systemic lupus erythematosus
ssDNA: Single-stranded DNA
STAT: Signal transducers and activators of transcription
STING: Stimulator of interferon genes
TBK1: TANK-binding kinase 1
TCR: T cell receptor
TLR: Toll-like receptor
Treg: T regulaory cell
TSCC: Tongue squamous cell carcinoma
Tyk2: Tyrosine kinase 2
VEGF: Vascular endothelial growth factor
VEGFR2: Vascular endothelial growth factor receptor 2
WIPI2: WD repeat domain phosphoinositide-interacting protein 2
